# Constitutive STAT3 Phosphorylation in Circulating CD4^+^ T Lymphocytes Associates with Disease Activity and Treatment Response in Recent-Onset Rheumatoid Arthritis

**DOI:** 10.1371/journal.pone.0137385

**Published:** 2015-09-09

**Authors:** Krista Kuuliala, Antti Kuuliala, Riitta Koivuniemi, Suvi Oksanen, Mari Hämäläinen, Eeva Moilanen, Hannu Kautiainen, Marjatta Leirisalo-Repo, Heikki Repo

**Affiliations:** 1 Bacteriology and Immunology, Helsinki University Hospital and University of Helsinki, Helsinki, Finland; 2 Rheumatology, Helsinki University Hospital and University of Helsinki, Helsinki, Finland; 3 Immunopharmacology Research Group, University of Tampere School of Medicine and Tampere University Hospital, Tampere, Finland; 4 Primary Health Care, Helsinki University Hospital and University of Helsinki, Helsinki, Finland; 5 General Practice, Helsinki University Hospital and University of Helsinki, Helsinki, Finland; 6 Unit of Primary Health Care, Kuopio University Hospital, Kuopio, Finland; SERGAS (Servizo Galego de Saude) and IDIS (Instituto de Investigación Sanitaria de Santiago), the NEIRID Lab, Research Laboratory 9, Santiago University Clinical Hospital. Santiago de Compostela, SPAIN

## Abstract

The aim of the present study was to examine constitutive signal transducer and activator of transcription 3 (STAT3) phosphorylation in circulating leukocytes as a candidate biomarker in rheumatoid arthritis (RA). 25 patients with recent-onset, untreated RA provided samples for whole blood flow cytometric determination of intracellular STAT3 phosphorylation, expressed as relative fluorescence units. The occurrence of constitutive STAT3 phosphorylation was evaluated by determining proportion of STAT3-phosphorylated cells among different leukocyte subtypes. Plasma levels of interleukin (IL)-6, IL-17 and IL-21 were measured by immunoassay, radiographs of hands and feet were examined and disease activity score (DAS28) was determined. Biomarkers were restudied and treatment response (according to European League Against Rheumatism) was determined after 12 months of treatment with disease-modifying antirheumatic drugs. At baseline, constitutive phosphorylation of STAT3 occurred in CD4^+^ T cells of 14 (56%) patients, CD8^+^ T cells of 13 (52%) patients, in CD19^+^ B cells of 7 (28%) patients, and in CD14^+^ monocytes of 12 (48%) patients. STAT3 phosphorylation levels of CD4^+^ T cells associated with DAS28, and those of all leukocyte subtypes studied associated with erosive disease. The presence of constitutive STAT3 phosphorylation in CD4^+^ T lymphocytes, pSTAT3 fluorescence intensity of CD4^+^ and CD8^+^ T cells and C-reactive protein (CRP) levels at baseline associated with good treatment response. In conclusion, constitutive STAT3 phosphorylation in circulating CD4^+^ T cells is common in recent-onset untreated RA and associates with good treatment response in patients characterized by high disease activity and the presence of systemic inflammation.

## Introduction

Rheumatoid arthritis (RA) is a chronic, heterogeneous inflammatory disease with autoimmune origin. The treatment options include disease-modifying antirheumatic drugs (DMARDs), biological DMARDs targeted against the actions of T cells, B cells, the inflammatory cytokines tumour necrosis factor (TNF) or interleukin (IL)-6, and small molecular inhibitors of intracellular kinases [1,2]. At present, there are virtually no tests that could reliably predict the individual disease course and response to the chosen treatment. Hence, it would be of great value to find novel biomarkers that help support the diagnosis, reveal the contribution of different immune cell types and activity of inflammatory processes, and predict the progression of the disease and the efficacy and suitability of the treatment strategies.

Conventionally, plasma/serum samples have been used in order to find helpful biomarkers for RA activity and progression [3–7]. However, levels of these marker candidates are often related to markers of inflammatory activity that are already in clinical use (most commonly to C-reactive protein (CRP) and erythrocyte sedimentation rate (ESR)), whereas their independent marker potential may remain limited [3,5,7]. Our recent approach is to study the activation of intracellular signaling pathways in leukocytes, using phosphospecific whole blood flow cytometry, as sign of leukocyte activation in inflammatory disease conditions [8–13]. The method is considered applicable for immune status determination before starting immune-modulating therapy in inflammatory diseases [14].

STATs (signal transducers and activators of transcription) are pivotal cytoplasmic transcription factors that become activated by phosphorylation and migrate into the nucleus. STAT activation usually takes place when extracellular stimuli, e.g. various cytokines and growth factors, bind to their cell surface receptors and trigger intracellular phosphorylation cascades [15–17]. STAT3 regulates critical cellular processes such as growth, survival and transcription of inflammatory genes [16–19]. Activation of STAT3 has been described in monocytes from synovial fluid [20] and in T cell infiltrate [21] in the joints of patients with RA, and results from experimental arthritis models support the concept that STAT3 promotes arthritis when activated in the joints [21,22]. We first got evidence that STAT3 phosphorylation can be linked to the activity of RA and the disease process when we screened, along with signaling profiles of patients with a history of reactive arthritis [11], patients with early RA. In these preliminary experiments, blood lymphocytes and monocytes from the patients with RA revealed constitutive STAT3 phosphorylation [23]. This prompted us to study constitutive STAT3 phosphorylation in subtypes of blood leukocytes of patients with recent-onset, untreated RA in relation to disease activity and treatment response in the present study.

The results show that constitutive STAT3 phosphorylation in blood leukocytes of patients with early DMARD-naïve RA associates with disease activity, plasma levels of proinflammatory cytokines IL-6, IL-17 and IL-21, and treatment response. The findings introduce STAT3 phosphorylation in circulating leukocytes, especially in CD4^+^ T lymphocytes, as a potential novel biomarker to predict treatment response in early RA.

## Materials and Methods

### Subjects

The study includes 25 patients diagnosed with RA at the Division of Rheumatology, Helsinki University Central Hospital, from April 2010 to October 2011. The patients fulfilled the American College of Rheumatology/European League Against Rheumatism (ACR/EULAR) 2010 classification criteria [24]. At baseline, the patients had received no DMARDs or oral corticosteroids. They did not take non-steroidal anti-inflammatory drugs regularly either before or after entering the study.

Laboratory and hospital personnel who did not have autoimmune diseases or immunosuppressive medication served as healthy reference subjects (n = 17).

The study protocol was approved by the Ethical Review Board of the Joint Authority for the Hospital District of Helsinki and Uusimaa, and written informed consent was obtained from each patient.

### Clinical evaluation

A comprehensive clinical and laboratory evaluation was undertaken at entry concomitant to blood sampling, and after follow-up time (median 12 months, range 6 to 18 months) to assess outcome. 66/68 joints were evaluated for swelling and pain, patient’s global assessment of disease activity was recorded on a 100 mm visual analogue scale, and laboratory measurements including ESR and serum CRP level were logged. Disease activity score using 28 joints (DAS28) was calculated [25]. Treatment response was evaluated using EULAR response criteria [26]. Postero-anterior radiographs of hands and feet were obtained at baseline and presence of erosions was evaluated by an experienced rheumatologist (MLR).

### Blood samples

A 4-ml blood sample was taken, at baseline and at follow-up, by venipuncture from the antecubital vein into a Falcon polypropylene tube (Becton Dickinson) supplemented with 400 μl of pyrogen-free acid citrate dextrose solution A (ACD-A, Baxter). Cells for flow cytometry were prepared within 3 hours of blood sampling. Plasma was separated from the remaining blood sample by centrifugation and frozen in aliquots at -80°C.

### Preparing leukocytes for flow cytometry

Blood samples were prepared according to protocol by Becton Dickinson [27]. All antibodies were also purchased from BD. The optimal antibody amounts and the compatibility of the antibodies with the permeabilization procedure were tested in preliminary experiments.

Briefly, three 100-μl aliquots of blood were put into polystyrene tubes (BD). The tubes were supplemented with of either a) anti-CD4-FITC/CD8-PE antibody (mouse anti-human IgG_1_, κ, clone SK3/SK1, 5 μl), b) anti-CD19-FITC (mouse anti-human IgG_1_, κ, clone SJ25C1, 9 μl), or c) anti-CD14-FITC (mouse anti-human IgG_2b_, κ, clone MᵩP9, 5 μl). Following 15-minute incubation at +37°C, the leukocytes were fixed and erythrocytes lysed by adding 1X Lyse/Fix buffer. After pelleting, leukocytes were washed with Stain Buffer and permeabilized by Perm Buffer III at -20°C for 30 min. Cells were pelleted and washed with Stain Buffer, after which all tubes were supplemented with anti-STAT3 (pSer727)-Alexa Fluor 647 antibody (mouse anti-human IgG_1_, clone 49/p-Stat3, 5 μl), and tubes a and b also with anti-CD3-PerCP antibody (mouse anti-human IgG_1_, κ, clone SK7, 9 μl), in 100 μl of Stain Buffer. Following incubation at room temperature protected from light for 20 min, the cells were washed in Stain Buffer and resuspended in 300 μl of Stain Buffer. The samples were kept on ice for a maximum of 4 hours until flow cytometric acquisition.

### Flow cytometry

Flow cytometric data were acquired on FACSCantoII flow cytometer and analyzed with FACSDiva software (BD BioSciences, San Jose, CA), as described previously [9]. CD4^+^ and CD8^+^ T lymphocytes, CD19^+^ B lymphocytes and CD14^+^ monocytes were identified and pSTAT3 fluorescence intensity of the respective histograms was determined as relative fluoresence units (RFU). The proportion of pSTAT3 positive (pSTAT3^+^) cells in the patients’ samples was determined as described previously [9,10]. In brief, a marker was set on each histogram from a healthy subject’s sample obtained within a week from that of a patient’s sample so that it encompassed less than but as close as possible to 5% of the events, and the markers were then copied to the respective histograms from the patient’s sample. If a marker encompassed more than 5% of the events, the patient was said to be pSTAT3^+^ for the corresponding leukocyte subset. Each healthy subject studied served as a reference for one to three patients. The use of the proportion of pSTAT3^+^ cells, as compared to that of fluorescence intensity values, aids to minimize the variation related to phosphospecific flow cytometry [28] and provides a more sensitive and illustrative way to reveal constitutive phosphorylation occurring within leukocyte populations of the patients.

### Plasma IL-6, IL-17 and IL-21 determination

Plasma levels of IL-6, IL-17 and IL-21 were determined by enzyme-linked immunoassay (ELISA) by using the reagents from eBioscience Inc (San Diego, CA, USA). The detection limits and inter-assay coefficients of variation were 0.39 pg/ml and 1.9% for IL-6, 0.98 pg/ml and 6.3% for IL-17, and 15.6 pg/ml and 5.7% for IL-21. In the immunoassays, possible influence of rheumatoid factor and related compounds present in the patient samples was tested by adding commercially available heterophilic blocking agent HeteroBlock (Omega Biologicals, Bozeman, MT, USA) in the samples in increasing concentrations up to 600 μg/ml as recommended by Todd *et al*. [29]. No influence on IL-6 concentrations was found while when measuring IL-17 and IL-21 concentrations, the samples were treated with 300 μg/ml and 150 μg/ml of HeteroBlock blocking reagent, respectively.

### Statistical analysis

The data are presented as means with standard deviations (SD), medians with interquartile range (IQR), or counts with percentages. Statistical comparisons were made by using bootstrap type t-test, permutation test, Mann-Whitney U test with exact p values and permutation type analysis of co-variance. However, as sample size was small and some variables were skewed, exact and resampling-based (Monte Carlo permutation and bootstrap) methods were used to achieve significance level and 95% confidence intervals (CI). Correlations were estimated by Spearman’s correlation coefficient method. Exact logistic regression models [30] were used to investigate factors related to treatment response. Receiver operating characteristic (ROC) curves were constructed to determine the predictive ability of baseline markers that corresponds to the EULAR response, with bias corrected bootstrap CI. Stata 13.1 (StataCorp LP, College Station, TX, USA) statistical package was used for the analyses.

## Results

### Clinical course and treatment

A total of 25 recently diagnosed DMARD naïve RA patients participated in the study (Table 1). After blood sampling 24 patients started DMARD (methotrexate, sulfasalazine, hydroxychloroquine) therapy according to the national guidelines [31] and EULAR recommendations [32]: 10 patients (40%) one DMARD, 6 patients (24%) a combination of 2 DMARDs, 7 patients (28%) a combination of 3 DMARDs, and 1 patient (4%) received a biological DMARD (tocilizumab) and methotrexate. In addition, 10 patients (40%) started a course of low-dose (≤ 10 mg/day) oral prednison. During follow-up the drug treatment was modified, targeting to remission, in line with the national and EULAR recommendations [31,32]. At follow-up, 22 of 25 patients (88%) were on DMARDs and 4 patients (16%) received oral prednison. The EULAR treatment response was good in 17 patients (68%), moderate in 2 patients (8%), and 6 patients (24%) did not respond (Table 1).

**Table 1:** Baseline characteristics according to EULAR response at follow-up.

Baseline variables	EULAR response at follow-up		p value
	No or moderate, N = 8	Good, N = 17	
**Demographics**			
Number of women, %	8 (100)	10 (59)	0.057
Age, years, mean (SD)	45 (15)	51 (15)	0.38
Duration of symptoms, months, mean (SD)	15 (13)	18 (21)	0.74
RF positive, n (%)	7 (88)	13 (76)	0.99
ACPA positive, n (%)	7 (88)	14 (82)	0.99
**Measures of disease activity**			
DAS28, mean (SD)	2.98 (1.61)	4.31 (1.37)	0.042
Erythrocyte sedimentation rate, mm/h, mean (SD)	14 (14)	30 (24)	0.097
Serum C-reactive protein, mg/l, mean (SD)	3 (1)	24 (28)	0.012
Number of swollen joints, 0–66, mean (SD)	8 (5)	9 (6)	0.69
Number of tender joints, 0–68, mean (SD)	6 (4)	9 (5)	0.19
Patient’s global assessment, 0-100mm VAS, mean (SD)	44 (31)	44 (17)	0.98
**Radiography**			
Patients with erosions, hands or feet, n (%)	1 (12)	5 (29)	0.62
**Plasma cytokine levels** *			
IL-6, pg/ml, median (min-max)	4.6 (1.8–126.4)	9.3 (1.8–85.7)	0.16
IL-17, pg/ml, median (min-max)	4.3 (2.7–9.8)	7.2 (1.0–21.6)	0.12
IL-21, pg/ml, median (min-max)	264 (121–598)	279 (39–763)	0.72
**pSTAT3** †			
*CD4* ^+^ *T cells*			
RFU, mean (SD)	596 (106)	752 (149)	0.011
pSTAT3^+^, n (%)	1 (12)	13 (76)	0.007
*CD8* ^+^ *T cells*			
RFU, mean (SD)	546 (95)	627 (103)	0.038
pSTAT3^+^, n (%)	2 (25)	11 (65)	0.097
*CD19* ^+^ *B cells*			
RFU, mean (SD)	442 (88)	432 (115)	0.83
pSTAT3^+^, n (%)	2 (25)	5 (29)	0.99
*CD14* ^+^ *monocytes*			
RFU, mean (SD)	838 (179)	933 (289)	0.41
pSTAT3^+^, n (%)	4 (50)	8 (47)	0.99

Abbreviations: ACPA = anti-citrullinated protein antibody, DAS28 = 28 joint Disease Activity Score, RF = rheumatoid factor, RFU = relative fluorescence unit, VAS = visual analogue scale.

*Median (min-max) values for healthy reference subjects (n = 17); IL-6: 1.2 (0.8–2.9) pg/ml, IL-21: 151 (39–467) pg/ml, IL-17: 4.5 (1.0–19.2) pg/ml.

^†^Mean (SD) values for healthy reference subjects (n = 17); CD4^+^ T cells: 638 (122) RFU, CD8^+^ T cells: 596 (114) RFU, CD19^+^ B cells: 463 (89) RFU, CD14^+^ monocytes: 829 (443) RFU.

### pSTAT3 positivity (pSTAT3^+^) associates with disease activity and treatment response

**Fig 1:**
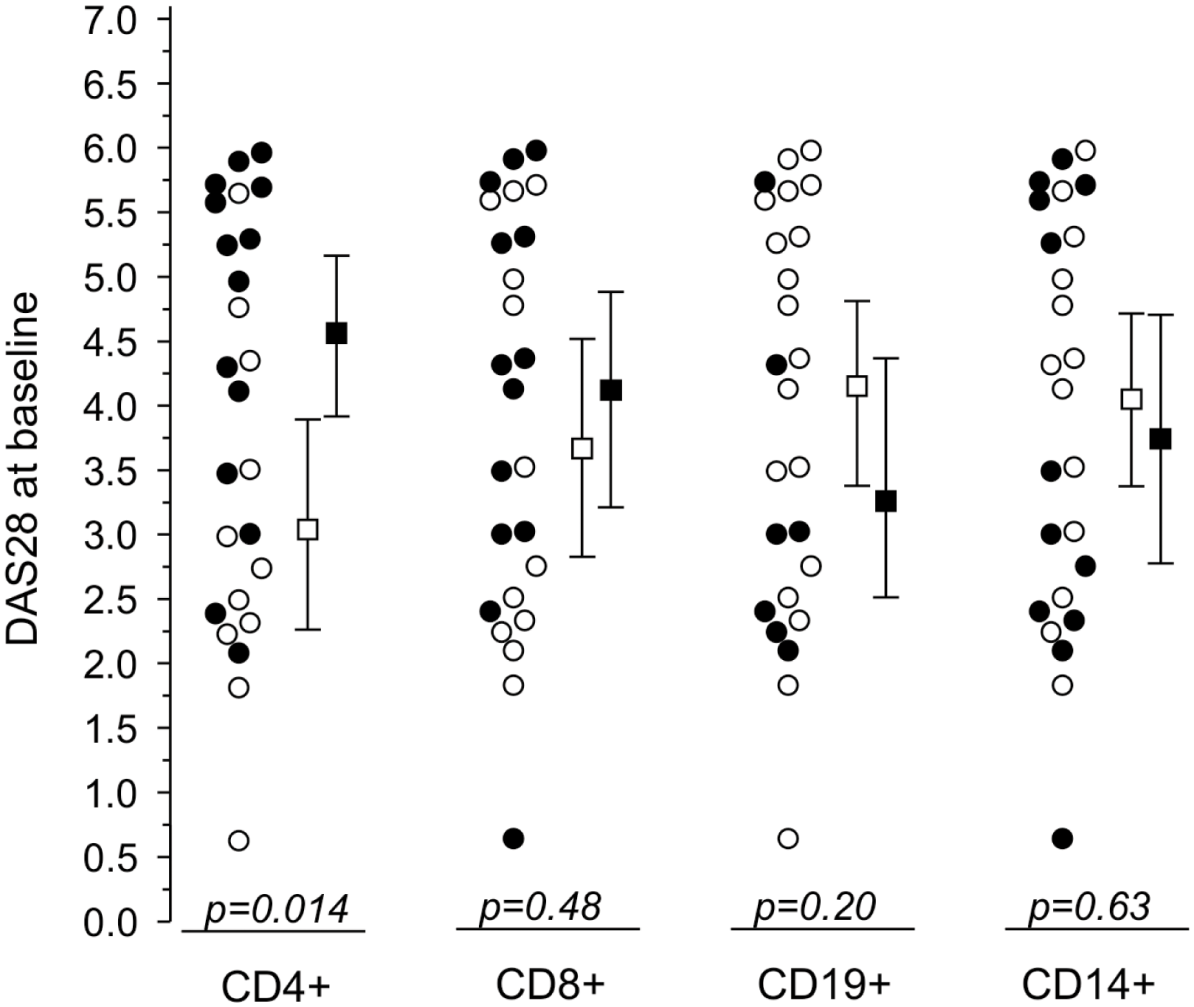
DAS28 scores (mean with 95% CI) at baseline according to baseline pSTAT3 positivity (pSTAT3^+^) of CD4^+^ and CD8^+^, CD19^+^, and CD14^+^ leukocytes. Closed symbols denote pSTAT3^+^ patients and open symbols pSTAT3^-^ patients. p values denote significance between pSTAT3^+^ and pSTAT3^-^.

At baseline, constitutive STAT3 phosphorylation was commonly found in circulating leukocytes from patients with recent-onset RA: CD4^+^ T cells from 14 patients (56%), CD8^+^ T cells from 13 patients (52%), CD19^+^ B cells from 7 patients (28%), and CD14^+^ monocytes from 12 patients (48%) were pSTAT3^+^. Interestingly, DAS28 score was associated with pSTAT3^+^ CD4^+^ T cells (Fig 1), suggesting an association between constitutive STAT3 phosphorylation in CD4^+^ T lymphocytes and disease activity in early RA.

Six of the 25 patients had erosive disease at baseline. Interestingly, all those six expressed pSTAT3^+^ CD8^+^ T cells whereas there were no erosions in patients with pSTAT3^-^ CD8^+^ T cells (p = 0.02). This finding suggests that early erosive RA is associated with activation of cytotoxic T lymphocytes.

During the follow-up time, DAS28 score decreased significantly more in patients in whom CD4^+^ or CD8^+^ T cells were pSTAT3^+^ at baseline than in those in whom they were not: the mean ratio of the change in DAS28 (follow-up adjusted for baseline DAS28) was 3.24-fold (95% CI 1.28 to 16.14) higher in patients with pSTAT3^+^ CD4^+^ T cells than in patients with pSTAT3^-^ CD4^+^ T cells, and 1.61-fold (95% CI 1.09 to 2.56) higher in patients with pSTAT3^+^ CD8^+^ T cells than in patients with pSTAT3^-^ CD8^+^ T cells.

**Fig 2:**
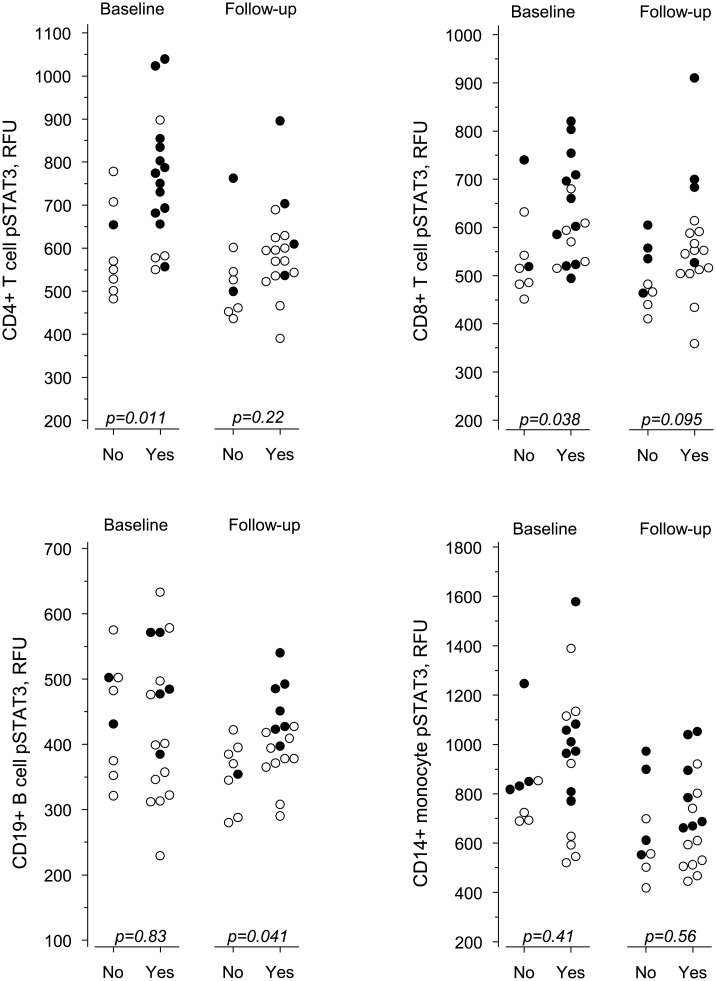
The pSTAT3 fluorescence intensity of lymphocyte subsets and monocytes at baseline and at follow-up according to EULAR treatment response. Yes denotes good response, No denotes no or moderate response, and p values indicate significance between the two groups. Closed symbols denote pSTAT3^+^ patients and open symbols pSTAT3^-^ patients.

To further determine the actual improvement during follow-up, EULAR response criteria were used. Patients with good response were categorized as good responders and patients with moderate or no response as non-responders. Thirteen out of the 14 patients with pSTAT3^+^ CD4^+^ T cells at baseline reached a good EULAR treatment response and one patient was a non-responder (Table 1, Fig 2) indicating that presence of pSTAT3^+^ CD4^+^ T cells in early RA may serve as predictor of good treatment response. Of the 11 patients not having pSTAT3^+^ CD4^+^ T cells at baseline, seven were non-responders to therapy and four had a good response (Fig 2, baseline data).

### pSTAT3 fluorescence intensity associates with disease activity, systemic inflammation and treatment response

As presented in the previous paragraphs, the presence of pSTAT3^+^ leukocytes was associated with disease activity and treatment response in early RA. Therefore we aimed to investigate if the intensity of pSTAT3 fluorescence (depicting the magnitude of STAT3 phosphorylation) is also a determinant of disease activity and treatment response in our patients proving evidence of quantitative association and potential causality.

pSTAT3 fluorescence intensity in CD4^+^ T lymphocytes correlated positively with DAS28 score at baseline (Table 2). Furthermore, pSTAT3 fluorescence intensity of each leukocyte subtype showed a positive correlation with erosion score, CD8^+^ T cells showing the strongest correlation (Table 2).

**Table 2:** Correlations between baseline characteristics and pSTAT3 fluorescence intensity of lymphocyte subsets and monocytes at baseline.

	CD4^+^	CD8^+^	CD19^+^	CD14^+^
	r (95% CI)	r (95% CI)	r (95% CI)	r (95% CI)
**Age**	0.26 (-0.17 to 0.65)	0.20 (-0.22 to 0.62)	-0.01 (-0.47 to 0.45)	-0.01 (-0.42 to 0.42)
**Duration of symptoms**	-0.28 (-0.60 to 0.10)	-0.03 (-0.41 to 0.36)	0.33 (-0.03 to 0.70)	0.06 (-0.31 to 0.43)
**RF**	0.21 (-0.11 to 0.52)	0.03 (-0.30 to 0.37)	0.10 (-0.27 to 0.48)	0.37 (-0.02 to 0.67)
**DAS28**	0.42* (0.10 to 0.77)	0.21 (-0.21 to 0.65)	-0.02 (-0.45 to 0.42)	0.26 (-0.18 to 0.69)
**Erythrocyte sedimentation rate**	0.30 (-0.06 to 0.66)	0.08 (-0.30 to 0.47)	-0.26 (-0.70 to 0.18)	0.10 (-0.34 to 0.53)
**Serum C-reactive protein**	0.29 (-0.08 to 0.66)	0.20 (-0.16 to 0.55)	-0.27 (-0.69 to 0.16)	0.09 (-0.33 to 0.51)
**Number of swollen joints, 0–66**	0.23 (-0.18 to 0.63)	0.05 (-0.39 to 0.49)	0.19 (-.26 to 0.63)	0.28 (-0.20 to 0.75)
**Number of tender joints, 0–68**	0.25 (-0.14 to 0.63)	0.18 (-0.25 to 0.61)	0.26 (-0.13 to 0.65)	0.33 (-0.10 to 0.76)
**Patient’s global assessment, 0-100mm VAS**	-0.01 (-0.44 to 0.42)	-0.18 (-0.62 to 0.25)	-0.05 (-0.45 to 0.35)	-0.12 (-0.51 to 0.26)
**Erosion score** ^†^	0.44* (0.14 to 0.73)	0.62*** (0.40 to 0.85)	0.55** (0.28 to 0.82)	0.42* (0.13 to 0.71)
**Plasma IL-6**	0.45* (0.07 to 0.84)	0.07 (-0.38 to 0.52)	0.13 (-0.28 to 0.53)	0.41* (0.08 to 0.73)
**Plasma IL-17**	0.33 (-0.04 to 0.71)	0.26 (-0.19 to 0.71)	0.42* (0.04 to 0.80)	0.40 (-0.01 to 0.80)
**Plasma IL-21**	0.40* (0.06 to 0.74)	0.48* (0.17 to 0.80)	0.64*** (0.32 to 0.96)	0.68*** (0.48 to 0.89)

Abbreviations: CI = confidence interval, r = Spearman correlation coefficient, for other abbreviations see Table 1.

^†^Number of eroded joints in radiographs of hands and feet.

*p<0.05

**p<0.01

***p<0.001.

Circulating levels of CRP and proinflammatory cytokines, such as IL-6, are considered to serve as markers of systemic inflammation. At baseline, pSTAT3 fluorescence intensity of CD4^+^ T cells and monocytes correlated positively with IL-6 level, that of B cells correlated with IL-17 level, and that of each leukocyte subtype with IL-21 level (Table 2).

pSTAT3 fluorescence intensities in CD4^+^ and CD8^+^ T lymphocytes at baseline were associated with good treatment response (Table 1), as was serum CRP level. Multivariate exact logistic regression analysis was used to test for the independent explanatory value of pSTAT3 fluorescence intensity for treatment response. After adjusting for CRP level, pSTAT3 fluorescence intensity in CD4^+^ T cells statistically significantly predicted treatment response but that in CD8^+^ T cells did not.

During the follow-up time, pSTAT3 fluorescence intensity generally decreased (Fig 2). The decrease was significant among good responders in CD4^+^ T cells (p = 0.01) and CD14^+^ monocytes (p = 0.02). Also, there was a significant decrease in ESR (p<0.001), CRP (p<0.001), IL-6 (p<0.001), IL-17 (p<0.001) and IL-21 levels (p = 0.04) among good responders and in IL-17 levels among non-responders (p = 0.01). At follow-up, the pSTAT3 fluorescence intensities in CD4^+^ T cells and CD14^+^ monocytes and as well as the levels of ESR, CRP, IL-6, IL-17 and IL-21 of good responders were close to those of non-responders (Table 3).

**Table 3:** Erythrocyte sedimentation rate and levels of C-reactive protein and cytokines at follow-up according to EULAR response.

Variables	EULAR response at follow-up		p value
	No or moderate, N = 8	Good, N = 17	
**Erythrocyte sedimentation rate, mm/h, mean (SD)**	14 (3)	6 (5)	0.042
**Serum C-reactive protein, μg/ml, mean (SD)**	5 (6)	2 (1)	0.28
**IL-6, pg/ml, median (min-max)**	4.2 (1.7–136.6)	3.6 (1.9–13.6)	0.83
**IL-17, pg/ml, median (min-max)**	2.0 (1.9–2.3)	2.3 (1.9–6.7)	0.043
**IL-21, pg/ml, median (min-max)**	268 (54–816)	145 (31–579)	0.24

### The ability of cytokines, pSTAT3 fluorescence intensity, and C-reactive protein to predict EULAR treatment response

**Fig 3:**
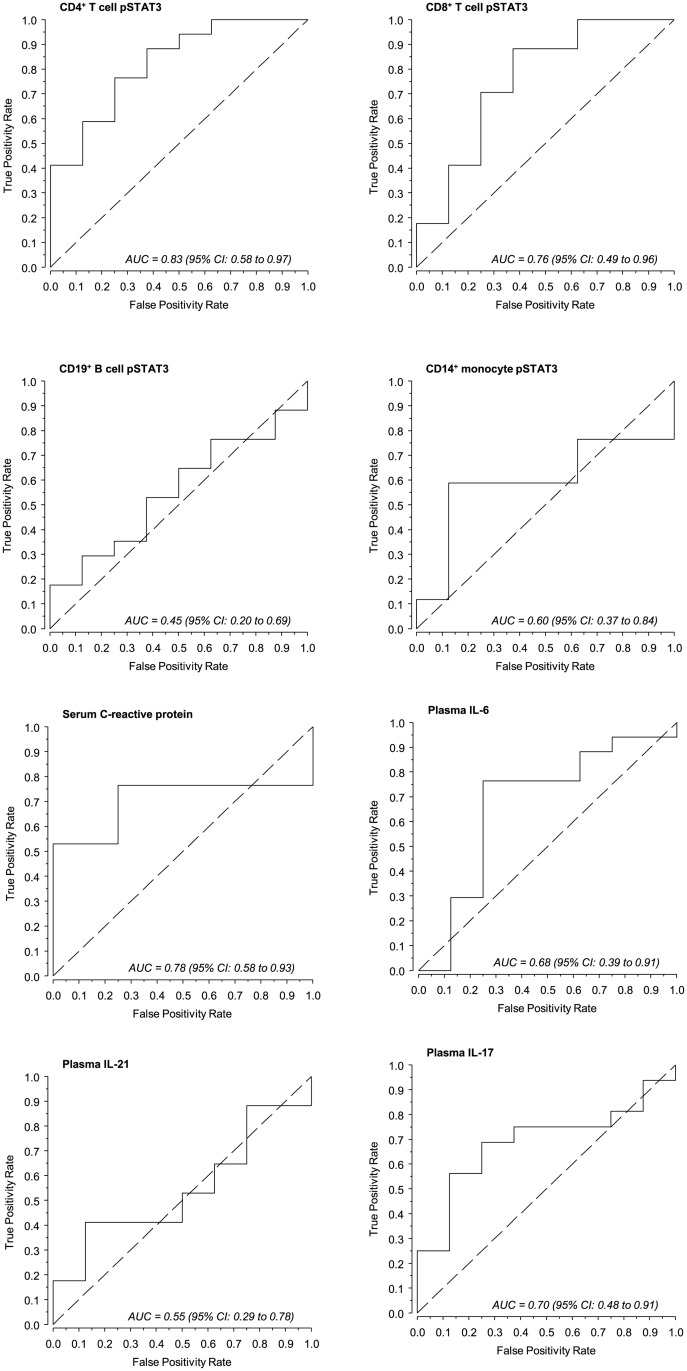
Predictive ability of pSTAT3 fluorescence intensity of leukocyte subsets, C-reactive protein and cytokines for EULAR treatment response. Area under the curve (AUC) values were determined for the baseline markers presented in Table 1. The dashed lines denote no information line.

To evaluate the ability of pSTAT3 fluorescence intensity to predict the EULAR response, ROC curves were generated (Fig 3). The AUC values of pSTAT3 fluorescence intensities of CD4^+^ and CD8^+^ T cells were close to the AUC of CRP and slightly higher than those of IL-6 and IL-17.

## Discussion

The results show that STAT3 is frequently phosphorylated in circulating leukocyte subsets of patients with recent-onset untreated RA. Our findings confirm the results of previous reports [23,33] and extend them by showing that constitutive STAT3 phosphorylation of CD4^+^ T cells identifies a subgroup of RA patients characterized by high disease activity, as determined by DAS28, presence of systemic inflammation, as determined by plasma CRP level, and good EULAR response to treatment with DMARDs. Constitutive phosphorylation of STAT3 in CD4^+^ T cells was recently found to occur in patients with undifferentiated arthritis in whom it served as a biomarker of progression to RA [34]. If this finding and ours are confirmed by other research groups, constitutive STAT3 phosphorylation may aid in developing treatment strategies for individual patients with undifferentiated arthritis and recent-onset untreated RA.

At present, constitutive STAT3 phosphorylation of the RA patients’ CD4^+^ T cells is considered to be driven by IL-6 [33,34]. In accordance with this, our results showed a positive correlation between plasma IL-6 levels and pSTAT3 fluorescence intensity in CD4^+^ T cells and monocytes at baseline. IL-6, a multifunctional cytokine, is associated with several molecular and cellular characteristics of inflammation in RA [35]. Recent studies have further brought up the roles of IL-6 in systemic inflammation in RA, e.g. cytokine and chemokine production of peripheral blood mononuclear cells (PBMC) [36], and the potential of plasma IL-6 levels to serve as a biomarker of structural damage in the joints during the first years of RA (independently of CRP levels) [37]. Also, as IL-6/STAT3 signaling has been shown to promote the differentiation of naïve T cells into B cell helper T cells [38], the activation of STAT3 by IL-6 in CD4^+^ T cells may expand cellular activation to B cells to enhance the development of RA [37]. A novel finding in the present study was that plasma levels of IL-21 were positively correlated with pSTAT3 fluorescence intensity in all leukocyte subtypes studied. IL-21 modulates, for example, immune responses of both T cells and B cells [38,39]. Our results also showed that plasma level of IL-17 correlates with pSTAT3 fluorescence intensity in B cells. IL-17 is the major contributor in developing the Th17 type of immune response and considered to play numerous roles in the pathogenesis of RA [40]. Like *IL21*, *IL17* is a direct target of pSTAT3 [15]. Clearly, the molecular relationships between constitutive STAT3 phosphorylation in circulating leukocyte subsets and plasma cytokine levels described in the present study, and systemic inflammation remain to be elucidated. In addition to altered cytokine milieu in peripheral blood, constitutive STAT3 phosphorylation in RA may involve aberrations in the expression or activation of intracellular kinases or negative regulators upstream of STAT3 or in transcriptional activity of *STAT3*. In this context it is of note that elevated levels of STAT3 mRNA in circulating monocytes and CD3^+^ T cells [33] and downregulation of suppressor of cytokine signaling 3 (a negative STAT3 pathway regulator) in CD4^+^ T cells [41] have been reported in RA patients.

We observed radiological erosions in six of 25 patients with recent-onset untreated RA at the time of diagnosis. A novel finding was that the number of eroded joints correlated with pSTAT3 fluorescence intensity in all leukocyte subtypes studied, and the correlation was strongest for CD8^+^ T cells. This may reflect the influence of IL-21, as IL-21 has been reported to be up-regulated in the synovium and synovial fluid of patients with RA and to enhance osteoclastogenesis in vitro [42]. In this context it is of note that serum levels of granzyme B, an acidic protease expressed by cytotoxic T lymphocytes, have been reported to be increased in arthritis and positively correlated with erosive RA [4], and that IL-21 increases its expression in CD8^+^ T cells [43]. Also, additional cytokines may be able to activate both STAT3 activation and granzyme B expression in CD8^+^ T cells [44]. Altogether, regardless of the mediating mechanism, granzyme B could be one of the factors connecting pSTAT3^+^ CD8^+^ T cells and presence of erosions.

Another novel finding in the present study was that pSTAT3 positivity in CD4^+^ T cells in recent-onset untreated RA is in relationship with successful response to therapy with DMARDs, as evaluated by EULAR response and effectual decrease in DAS28 after one year of treatment. Furthermore, during the follow-up time, STAT3 phosphorylation level in CD4^+^ T cells and monocytes decreased significantly among good responders, whereas there was no statistical difference between baseline and follow-up STAT3 phosphorylation levels among non-responders. Accordingly, Anderson et al. [34] have also reported that DMARD treatment depressed constitutive STAT3 phosphorylation. Even though the rationale of the result warrants further studies, an explanation may be provided by the molecular effects of DMARDs that directly counteract STAT3 activation-related phenomena. In this context it is noteworthy that activated STAT3 can activate over 3000 genes in CD4^+^ T cells [18], including genes involved in cell adhesion and migration to the joints [45,46], as well as genes important for proliferation and cell cycle regulation [17,18,47,48] analogously to malignant cells in which STAT3 is constitutively activated to promote prolonged functioning of dysregulated cells [15,16,47–50]. Considering DMARDs, sulfasalazine, for example, interferes with CD11b/CD18-mediated leukocyte recruitment [51] and chloroquine suppresses cell-cell adhesion mediated by β1 integrins [52]. Furthermore, considering that the levels of systemic inflammation biomarkers significantly decreased among good responders in our study, it is notable that methotrexate alone or in combination with sulfasalazine reduces circulating levels of IL-6 [53], and hydroxychloroquine inhibits IL-6, IL-17 and IL-22 production from PBMC [54]. Altogether, an interesting possibility introduced in the current study is that pSTAT3 positivity in peripheral blood CD4^+^ T cells could be a marker indicating a disease form of RA that is effectively alleviated by synthetic DMARDs.

Although constitutive STAT3 phosphorylation in CD4^+^ T cells of RA patients with recent-onset disease was clear in the present study and the previous studies [23,33,34], it was not evident in a recent study of RA patients refractory to DMARD therapy [55]. In the study by Isomäki et al. [33], 11/15 patients had long-standing RA. Because DMARD therapy depresses STAT3 phosphorylation, as shown previously [34] and in the present study, differences in DMARDs taken by the patients and disease duration may explain the discrepancy. Another explanation for discrepancy involves the methods used. To minimize *ex vivo* leukocyte activation, we and the two other groups [33,34] studied whole blood samples whereas Ortiz et al. [55] separated mononuclear cells from blood by density gradient centrifugation. Sample handling *ex vivo* readily promotes leukocyte activation [56–58], which may distort constitutive STAT phosphorylation data. Meticulous sample handling is a prerequisite for the study of constitutive STAT3 phosphorylation.

The present results show that constitutive STAT3 phosphorylation is common in circulating leukocytes of patients with recent-onset untreated RA, associates with active disease when present in CD4^+^ T cells, associates with presence of erosions when present in CD8^+^ T cells, and identifies a patient group designated by the presence of systemic inflammation and good response to treatment with synthetic DMARDs when present in CD4^+^ T cells. Optimally, the whole blood flow cytometric method we used may provide a rapid and easy way to recognize the RA patients in whom aberrant STAT3 signaling takes place. In the future, STAT3 phosphorylation status of leukocytes may, either alone or as part of a larger signaling profile, be determined for designing most beneficial and precisely targeted personalized treatment for patients with recent-onset RA.

## Conclusions

Constitutive STAT3 phosphorylation in circulating leukocytes is common in recent-onset untreated rheumatoid arthritis and identifies patients characterized by the presence of systemic inflammation and good EULAR response to DMARD treatment.

Measurement of intracellular STAT3 phosphorylation in circulating T lymphocytes may provide a novel predictive biomarker in RA and warrants further studies.
